# Does Dexmedetomidine Improve or Worsen Restless Leg Syndrome under Sedation: A Case Report and Extensive Review

**DOI:** 10.1155/2022/2447461

**Published:** 2022-09-05

**Authors:** Sandra Iskandar, Marina Souto Martins, Andrew Hudson, Jason G. Hirsch, Jonathan S. Jahr

**Affiliations:** ^1^University of California-Riverside Medical Student, Riverside, CA, USA; ^2^School of Medicine, University of Fortaleza, Brazil SA; ^3^David Geffen School of Medicine at UCLA, USA

## Abstract

**Background:**

Restless leg syndrome (RLS) is a common neurological condition that manifests as creeping, nonpainful urges to move lower extremities and is relieved with movements of the legs. RLS is associated with comorbidities such as gastric surgery, diabetes mellitus, uremia, and iron deficiency anemia, and it is misdiagnosed in many cases. Drugs like levodopa, ropinirole, pramipexole, cabergoline, and pergolide that target the dopaminergic system have been traditionally used to treat symptoms of RLS. *α*2-adrenoceptor (*α*2-AR) agonists, like clonidine and dexmedetomidine, have also been reported to show improvement of RLS symptoms during sedation. *Specific Aim*. This case report suggests that dexmedetomidine may have worsened RLS during sedation in a 71-year-old male with no prior diagnosis of RLS or reported symptoms. The patient had a procedure for right first metatarsophalangeal joint (MTPJ) fusion, with second digit proximal interphalangeal joint (PIPJ) arthrodesis, and flexor tendon transfer due to pain on walking and failing conservative therapy. He underwent intravenous sedation/monitored anesthesia care (MAC) with propofol, dexmedetomidine, and a peripheral regional block for intraoperative anesthesia and postoperative analgesia. During the surgery, the patient experienced continuous bilateral leg movement, unpredictable, and unrelated to surgical stimulation or level of consciousness within 5 minutes of administration of dexmedetomidine. The patient tolerated the procedure, and the unpredicted leg movement was managed by the surgeons intraoperatively.

**Conclusion:**

Although no previous literature exists and mechanisms are unclear, this case report hypothesizes that dexmedetomidine may contribute to worsening RLS symptoms.

## 1. Introduction

Restless leg syndrome (RLS) [1] is a relatively common condition affecting from 1% to 15% of the general population [2] and may be challenging to treat if present during moderate to deep sedation [3]. Dexmedetomidine has been reported to improve RLS during sedation [3]. We report a case where dexmedetomidine may have worsened RLS associated lower extremity movement during sedation and then present the current literature on RLS, and the case report is describing dexmedetomidine actions improving RLS during sedation. We comment that dexmedetomidine may exacerbate RLS under sedation and discuss mechanisms whereby this may occur.

## 2. Case Presentation

A 71-year-old man was scheduled for right first metatarsophalangeal joint (MTPJ) fusion, with second digit proximal interphalangeal joint (PIPJ) arthrodesis, and flexor tendon transfer due to pain on walking and failing conservative therapy. His extensive past medical history included hypertension (HTN), hypersensitivity lung disease (HLD), and chronic chest pain under investigation. He was also diagnosed with asthma and depression. He had a history of previous surgeries, without any reported problems related to anesthesia. His medications included metoprolol, atorvastatin, budesonide/formoterol, ipratropium, venlafaxine, albuterol, and clonidine. He had a smoking history for 3 years, which he quit during his twenties. Alcohol drinking habits were described as rare, and the patient denied cannabis or other substance use.

He did not have any finding during physical exam on preoperative anesthesia assessment, and for his preoperative laboratory work up, the only finding was an electrocardiogram (EKG) with sinus rhythm (SR) and a right bundle branch block (RBBB). The patient remained a candidate for intravenous sedation/monitored anesthesia care (MAC), with a peripheral regional block for intraoperative anesthesia and postoperative analgesia.

His baseline blood pressure (BP) and HR during the procedure were 156/92 mmHg and 91/min, respectively. His oxygen saturation (SpO2) was 96% while breathing room air. The patient received 20 milligram (mg) perineural infusion of mepivacaine 1.5%/mL during preoperative care. Additionally, after intravenous access was obtained, the patient received 20 micrograms (mcg) of dexmedetomidine and was placed on propofol infusion. He also received 30 mg of ketamine for multimodal anesthesia throughout the procedure.

His intraoperative course was complicated by the unanticipated event of almost continuous bilateral leg movement, unpredictable and unrelated to surgical stimulation or level of consciousness within 5 minutes of administration of dexmedetomidine. His depth of sedation was varied to deep sedation all the way to light sedation, with no improvement in symptoms of leg movements. The surgical team was able to continue surgery by placing the anesthetized foot in a padded restraint.

However, the nonoperative leg continued to move and caused challenges for both anesthesia and surgical teams. Discussion as to whether to convert to a general anesthetic occurred, but the surgeons believed they could complete the surgery safely and successfully, despite suboptimal conditions. The anesthesia team was concerned that a general anesthetic, with the patient's significant comorbidities, might prolong his postoperative course and even require a hospitalization.

The procedure was managed with varying the depth of sedation with the propofol infusion and later, addition of ketamine, in an attempt to provide additional sedation, improve the surgical operating conditions, and decrease leg movement. In the end, the patient tolerated the procedure under sedation and the surgeons managed to deal with the unpredictable leg movements, and no noticeable improvements occurred with either propofol at varying depths of sedation, dexmedetomidine, or later the case, ketamine. The patient did not receive any ondansetron or metoclopramide as antiemetics.

The surgery lasted three hours and seventeen minutes. The patient was transferred to the postoperative anesthetic care unit (PACU) for further observation. His postoperative course was uneventful and was discharged to a 23-hour observation unit and discharged home the next day without untoward events, unaware of his RLS history.

## 3. Discussion

Restless leg syndrome (RLS) or Willis-Ekbom disease is a chronic movement disorder that manifests as creeping, nonpainful urges to move lower extremities and is relieved with movements of the legs. The disorder has diurnal patterns, and it manifests during sleep or at rest. In the United States, there are more than three million cases per year [1]. Diagnosis of RLS is often missed, and patients endure their symptoms for years without treatment [2]. Therefore, taking a detailed patient history along with polysomnography studies is key to diagnosing the syndrome. Another related movement disorder is periodic limb movement disorder (PLMD) which manifests in repetitive and jerking movements during sleep. Even though RLS is a sensory phenomenon, PLMD is often associated with RLS.

### 3.1. Etiology

RLS can be broken down into two types: primary and secondary. Primary can be further subcategorized as being either idiopathic or familial in origin. Familial restless leg syndrome tends to occur at younger than 45 years of age [1]. The inheritance pattern of familial cases follows an autosomal dominant pattern with multiple loci [4, 5]. Years of research into the genetics behind RLS have revealed the genetic heterogeneity of the condition leading RLS to be considered a complex genetic disease [6]. Secondary disease is associated with comorbidities such as gastric surgery, diabetes mellitus, uremia, and iron deficiency anemia [4, 7]. Varied responses to treatment have been seen depending on the etiology of the disease, making it imperative to distinguish the cause of patients' symptoms [4]. Some studies suggest increasing prevalence with age [8], and others show that the cardiovascular and metabolic changes that occur with age are related to RLS [9].

RLS is associated with reduced intracortical inhibition rather than a specific lesion in the central nervous system (CNS) [4]. RLS symptoms seem to result from abnormal spinal sensorimotor integration at the spinal cord level and abnormal central somatosensory processing. This leads to increased sensory activation at the level of the spinal cord and decreased cortical inhibition of unwanted actions. Histological and functional imaging data together suggest that RLS exhibits a “hyperdopaminergic” presynaptic state characterized by increased synthesis and release of dopamine that is balanced by a “hypodopaminergic” postsynaptic state with decreased D2 receptor expression, though it remains unclear to what extent the pre and postsynaptic changes in RLS are primary, secondary, or compensatory [10].

### 3.2. Presentation

Patients present with nonpainful, irresistible urges to move their legs. Patients report their symptoms as a pulling, crawling, itching, or stretching sensation in the calves that happen at rest and is improved with leg movement. Studies show that the incidence of RLS is twice as high in women compared to men, which might suggest that hormones play a role in RLS [11].

### 3.3. Treatment

Drugs from various classes have been used off-label to treat the symptoms of RLS over the years. Multiple drugs that target the dopaminergic system have been used to treat RLS, which supports the hypothesis that central dopamine abnormalities play a significant role in the pathophysiology of RLS [12]. Double blind, placebo controlled studies demonstrated effectiveness of carbidopa/levodopa, propoxyphene, dopamine agonists, and pramipexole in reducing RLS symptoms [13–15]. Evidence from an evidence-based review by the International Parkinson and Movement Disorder Society found levodopa, ropinirole, pramipexole, cabergoline, pergolide, and gabapentin all are efficacious in treating RLS. However, augmentation, an iatrogenic worsening of RLS symptoms following treatment with dopaminergic agents, has been recognized as an important issue in the management of RLS [16]. Other studies have shown that benzodiazepines and baclofen are clinically effective in treating RLS [17, 18].

Gabapentin was shown to be efficacious in treating the symptoms of RLS. One study found that 600 mg of gabapentin enacarbil was efficacious for Japanese patients up to 12 weeks [16]. There are no major safety concerns with gabapentin. Side effects are common, and older patients may experience dizziness, somnolence, and peripheral edema [12]. These sedative side effects of gabapentin might suggest using the drug before or during sleep.

Clonidine has been reported in multiple studies as showing effective results in treating RLS. One study reported that the use of clonidine (mean = 0.05 mg/day) is useful in patients who experience delayed sleep onset due to leg sensations [19]. The study showed that clonidine decreased rapid movement sleep pattern (REM) sleep and increased REM latency, but did not change total sleep duration, stage 1 and 2 sleep, sleep efficiency, awakenings, arousals, or periodic limb movements in sleep. Another double-blind study conducted in 20 patients with chronic renal failure showed complete relief of RLS symptoms in 8 out of 10 patients upon treatment with clonidine for 3 days. Whereas only 1 out of 10 patients has mild alleviation in RLS symptoms in the placebo group [20].

Dexmedetomidine is a full *α*2-adrenoceptor (*α*2-AR) agonist that is similar to the partial *α*2-adrenoceptor agonist, clonidine. It is used in several clinical settings in view of its diverse actions which include sedation, analgesia, anxiolysis, perioperative sympatholysis, cardiovascular stabilizing effects, reduced anesthetic requirements, and preservation of respiratory function [21]. Dexmedetomidine is 8 to 10 times more selective towards *α*2-AR than clonidine [22]. Binding to the different subtypes of *α*2-AR allows dexmedetomidine to exert its diverse effects. Dexmedetomidine may cause decreasing the heart rate and vasoconstriction of the peripheral vasculature [23]. Dexmedetomidine also exerts a biphasic effect on the blood pressure by binding to two different subtypes of adrenergic receptors (AR). Dexmedetomidine evokes a short hypertensive phase mediated by its binding to *α*-2B adrenergic receptors followed by a hypotensive phase by binding to *α*-2B adrenergic receptors. Dexmedetomidine binds to *α*2A-AR subtype of *α*2-AR causing transient hypertension and to *α*2B-AR subtype leads to hypotension [24]. Dexmedetomidine causes its sedative effects by acting on the *α*2A-AR in the Locus ceruleus in the brain stem [23].

In addition, the adrenergic effects of dexmedetomidine produces antinociceptive effects and reduces release of the spinal neurotransmitter glutamate at the postsynaptic membrane [25, 26]. Of note, dexmedetomidine was recently shown to be effective in treating RLS exacerbation. In a case study report, a 22-year woman with suspected RLS was scheduled for bilateral sagittal split ramus osteotomy under general anesthesia [22]. During the surgery, general anesthesia included dexmedetomidine (0.2 *μ*g/kg/h), which was also administered at the same rate for postoperative sedation and analgesia. After the surgery, the patient began experiencing urges to move her legs described as “ants creeping on both ankles,” which resembled her previous RLS-like symptoms. The symptoms gradually became more severe (10/10) and spread to the upper extremities despite massaging the ankles. Dexmedetomidine infusion was increased from 0.2 to 0.4 *μ*g/kg/h for optimal bed rest and allowed her to sleep for 3 hours. Upon waking, the patient was able to walk and stretch and reported resolution of RLS symptoms, which suggested a role for dexmedetomidine in alleviating RLS exacerbations [22].

### 3.4. Proposed Mechanism of RLS after Dexmedetomidine Administration

The alpha 2 effect of dexmedetomidine in the dorsal horn would be expected to reduce the nociceptive sensory input related to RLS, which should reduce the motor response to the noxious sensations that normally trigger them.

In fact, we may have been observing not RLS but periodic limb movement disorder (PLMD), which is closely associated with RLS and produces spontaneous periodic leg movements during sleep. Even if the patient had RLS with PMLD, there is no immediately obvious explanation for symptom worsening with dexmedetomidine from a pharmacologic perspective as PLMD seems to be treated similarly to RLS, with dopaminergic agents, gabaergic agents, or gabapentinoids.

Our best explanation for how dexmedetomidine could worsen uncontrolled periodic movements centers on dopaminergic circuits; because these movements occurred regardless of sedation depth, it is unlikely that the movements were related to sensory phenomena as seen in RLS but were more analogous to the dyskinesias and athetosis in Parkinson's disease patients treated with dopaminergic agents. Interestingly, there is evidence that adrenergic agents can influence dyskinesias. Idazonax, an alpha-2 antagonist, has been shown to decrease dyskinesias in both PD humans and MPTP monkeys treated with L-dopa without worsening parkinsonian symptoms [27].

There are multiple typologies of movement disorders associated with L-dopa administration in PD patients [28]. Marconi et al. offered a proposal that, after a dose of L-dopa, the increasing secretion of dopamine in the striatum first begins to stimulate the dorsal putamen neurons before it is able to relieve parkinsonian symptoms, because this area usually has the greatest nigrostriatal hence may have the most hypersensitivity to dopamine (DA). This putamen area corresponds to cortico-striatal output to the lower extremity and also is rich in the D2 neurons that form the origin of the “indirect pathway” [29], resulting in the onset of dyskinesias and ballistic movements in the lower extremities. At least one study has demonstrated RLS patients have decreased 18F-dopamine uptake in the putamen with reduced D2 binding [30], and D2 receptor loss in the putamen was also demonstrated in RLS patients in an autopsy study [31].

In summary, while no literature exists that the authors are able to find, it may be that dexmedetomidine may worsen RLS. Mechanisms for this are unclear, but the authors present the following hypothesis that must be validated. Dexmedetomidine provides sedation without respiratory depression and analgesia. However, in order to produce these pharmacologic actions, it might deactivate the neural pathways that provide descending input to the peripheral nerves as well as centrally. This may in turn block the normal mechanism that RLS patients use to prevent leg movement consciously, although not completely prevent unintended leg movements. Blocking this descending neural pathway may explain why dexmedetomidine may worsen RLS in some patients, yet improve it in others, where the neural block for ascending pathways is sufficient to prevent leg movements.

## 4. Conclusion

We report a case of a 71-year-old patient with significant comorbidities and no history of RLS, who developed severe RLS symptoms after receiving sedation for a podiatric surgical procedure [32]. The symptoms were not improved by any therapy, and the surgical team was able to compensate for the movement, but the foot not undergoing surgery that did not have regional anesthesia was problematic in management. It may be that the dexmedetomidine exacerbated the symptoms of previously undiagnosed RLS or PLMD. Had this diagnosis been present preoperatively, given the intraoperative course, dexmedetomidine might have been avoided with possibly improved surgical conditions.
